# Why Are We Failing to Implement Imaging Studies with Radiolabelled New Molecular Entities in Early Oncology Drug Development?

**DOI:** 10.1155/2014/269605

**Published:** 2014-08-18

**Authors:** Azeem Saleem, Philip Murphy, Christophe Plisson, Michael Lahn

**Affiliations:** ^1^Imanova Ltd., Centre for Imaging Sciences, Imperial College Hammersmith Hospital, Du Cane Road, London W12 0NN, UK; ^2^GlaxoSmithKline Global Imaging Unit, Stockley Park West, 1-3 Ironbridge Road, Uxbridge, Middlesex UB11 1BT, UK; ^3^Early Phase Oncology Clinical Investigation, Eli Lilly Corporate Center, Building 31/4, 893 S. Delaware Street, Indianapolis, IN 46285, USA

## Abstract

In early drug development advanced imaging techniques can help with progressing new molecular entities (NME) to subsequent phases of drug development and thus reduce attrition. However, several organizational, operational, and regulatory hurdles pose a significant barrier, potentially limiting the impact these techniques can have on modern drug development. Positron emission tomography (PET) of radiolabelled NME is arguably the best example of a complex technique with a potential to deliver unique decision-making data in small cohorts of subjects. However, to realise this potential the impediments to timely inclusion of PET into the drug development process must be overcome. In the present paper, we discuss the value of PET imaging with radiolabelled NME during early anticancer drug development, as exemplified with one such NME. We outline the multiple hurdles and propose options on how to streamline the organizational steps for future studies.

## 1. Background

Access to and achieving therapeutic drug levels in the target tissue are a basic prerequisite for the successful development of a new molecular entity (NME). Conventionally, in drug development, plasma drug pharmacokinetics supplemented by preclinical data relating plasma to tissue pharmacokinetics is used as surrogate for target pharmacokinetics. However, increased realisation about interspecies differences and variable drug access in tumours and in sanctuary tissue sites, such as the brain, has led to the exploration of other methods that can provide confidence in tissue drug biodistribution and kinetics. One methodology that can provide such supportive information noninvasively is positron emission tomography (PET) imaging of radiolabelled NMEs. Radiolabelling of NME with a positron-emitting radionuclide to enable imaging does not change its biochemical properties and allows quantification of the NME at picomolar levels* in vivo* in tissue [1]. PET imaging continues to be used broadly in neurosciences to evaluate drug access to the target during early stages of clinical development [2, 3]. In oncology, PET imaging studies can provide valuable information on drug access to tumour tissue, which can be affected by a number of factors such as the P-glycoprotein (PgP) and breast cancer resistance protein (BCRP) [4, 5] drug efflux mechanisms and aberrant tumour vasculature [6] (Table 1). Despite this valuable tool, there have been no prospective studies which used PET imaging for early decision-making in oncology trials. Consequently, the full potential of such studies had not been fully harnessed.

NMEs can be radiolabelled with short-lived positron-emitting radioisotopes (e.g., carbon-11, half-life 20 mins; Fluorine-18, half-life 119 mins) or with longer half-lives (e.g., Zirconium-89, half-life 3.3 days; Iodine-124, half-life 100 hours). Since the longer half-life of Zirconium-89 (^89^Zr) and Iodine-124 (^124^I) matches the circulation half-lives of monoclonal antibodies (mAbs), theses isotopes have been used in the radiolabelling and evaluation of mAbs (Immuno-PET). Crucial developments including commercial availability of ^89^Zr and ^124^I, development and implementation of simplified radiolabelling techniques, and availability of radiolabelling protocols have allowed broad-scale clinical application of ^89^Zr- and ^124^I-immuno-PET in clinical mAb development studies [7]. However, such radiolabelling methods are not suited for other NMEs, which require development of molecule-specific radiochemistry. Moreover, the higher radiation doses associated with longer-lived PET radioisotopes limit its use in healthy volunteers and in performing repeat scans in the same subject.

In this paper, we have specifically focused on imaging studies of NMEs radiolabelled with short-lived radioisotopes. We will discuss the barriers in the implementation of PET studies, which currently limit the value of this tool. Using an example, we outline the logistics involved in conducting such studies. Finally, we propose ways to overcome potential barriers to streamline the performance of PET imaging studies with a particular emphasis on the conduct of such studies in the United Kingdom (UK).

## 2. PET Imaging Studies with Radiolabelled NMEs Are Ethically Justified and Provide Potential Savings in Drug Development

Because approximately 92% of oncology NMEs will not be approved [8], hundreds of patients receive limited or no additional benefit from participating in trials with NMEs. Incorporation of PET imaging studies in proof of concept studies, such as First-in-Human Dose (FHD) studies, is therefore a way to reduce attrition and is ethically justified, because it may help exclude ineffective NMEs early. As only 8% of oncology drugs reach the market, there has been an impetus to reduce late phase attrition by performing early proof of concept studies [9]. Typically phase I FHD studies are about a tenth (~£ 10 m) as expensive of a phase III study (~£ 100 m) [8]. Therefore, if PET studies are able to support a futility decision on a NME in a phase I proof of concept study, it will allow the drug developer to shift focus to other active NMEs. In the experience of the authors, the additional cost of a typical PET study in 6 to 10 patients including development of radiochemical synthesis methods, regulatory approvals, and data analysis can range between £ 0.5 and 1 million. Such studies may also reduce the number of subjects needed in a FHD study and thus offset the costs of the imaging study. All of the above may potentially lead to increased efficiency in drug development and lead to a significant reduction in the costs of drug development.

## 3. Pharmacological Information Obtained from Clinical PET Studies

PET is a highly sensitive technology allowing the quantification of picomolar tissue levels, thereby enabling studies to be performed at microdoses of the NME, typically defined as 1–100 *μ*g or <1/100 of the therapeutic dose. For instance, microdose studies with the radiolabelled topoisomerases I and II inhibitor, DACA, and [^11^C]DACA were performed at 1/1000th of the phase I starting dose [14], a significant advantage during early drug development. PET studies with radiolabelled NMEs can provide information on drug pharmacology and are especially valuable where standard pharmacokinetic evaluation cannot be extrapolated from plasma pharmacokinetic profile of the NME (e.g., brain). They can also provide key information on the drug pharmacokinetics when the NME has a complex structure and there is an uncertainty about the pharmacokinetic profile and its impact on the target site (e.g., antisense oligonucleotide; ASO). In the following we show the primary benefits of using PET. A potential caveat with PET pharmacokinetic studies with agents administered orally may result due to differences in bioavailability as PET studies are conducted with intravenous administration of the radiolabelled NME.

### 3.1. Direct Evidence of Drug Access to Tumour Tissue

PET studies can provide direct evidence of drug access to target and the relative uptake in tumours compared to normal tissue. PET studies with radiolabelled temozolomide ([^11^C]temozolomide) performed during early development demonstrated higher uptake of [^11^C]temozolomide in gliomas compared to normal brain tissue suggestive of a potentially advantageous therapeutic index [12]; currently, temozolomide is an approved and frequently used agent in the routine management of brain tumours.

### 3.2. Access to Sanctuary Sites

In contrast to brain tumours or metastases wherein the blood-brain-barrier is generally disrupted, drug access to normal brain remains a challenge. This is particularly an issue for monoclonal antibodies because their size does not allow them to cross an intact blood-brain-barrier and thereby fail to prevent CNS recurrences [27]. Although small molecules may have improved brain access due to their lower molecular weight, a number of factors influence the penetration of small molecules to the brain, such as lipophilicity, electric charge, affinity to active transport systems, or being a substrate compound for drug efflux mechanisms [28, 29]. Since there are limited models able to predict blood-brain-barrier penetration, a clinical PET study can provide direct confirmation. For example, a recent study of radiolabelled lapatinib, a small molecule targeting Her-1 and Her-2 tyrosine kinase, suggested that the drug did not cross an intact blood-brain-barrier [20]. Such an imaging study in small number of subjects can provide important insights into the lack of access to a sanctuary site, such as the brain, and thus avoid an expensive large trial or help design an appropriate confirmatory clinical study.

### 3.3. Evidence of Target Engagement and Drug Activity

Target engagement is crucial with the arrival of novel cytostatic and targeted agents especially since conventional structural imaging methods may not show a decrease in tumour size despite target engagement. For instance, target engagement of the anticancer agent LY2181308 as evidenced by the biodistribution of [^11^C]LY2181308 in tumours was proven through an associated decrease in tumour glucose metabolism measured by FDG-PET uptake [16]. Such direct and early evidence of target engagement could not have been substantiated in the absence of a decrease in tumour size on CT readout [16]. While PET studies using an appropriate pharmacodynamic radiotracer can provide an early readout of target engagement, they can also suggest resistance and/or sensitivity mechanisms, that is, discrepancy between tumour uptake and response to treatment. Other PET pharmacodynamic (PD) markers assess glucose metabolism (FDG), proliferation (Fluorothymidine), perfusion (water), angiogenesis (Fluciclatide) and hypoxia (fluoromisonidazole, HX4). The appropriate use of such a PET PD biomarker is based on the biology of the drug action and its presumed target.

### 3.4. Target Engagement for Patient Stratification

Potential differences in tumour phenotypic expression may be exploited to help stratify and in personalisation of therapy as elegantly exemplified by clinical PET studies with [^11^C]erlotinib, a tyrosine kinase inhibitor of epidermal growth factor receptor (EGFR). In patients with nonsmall cell lung cancer, higher uptake of [^11^C]erlotinib was observed in patients with exon 19 mutations compared to patients with wild type EGFR expression, potentially allowing for personalisation of therapy [21, 22]. There is an increasing interest in the radiolabelling tyrosine kinase inhibitor (TKI) to aid their clinical development as shown by studies with 7 of the 12 FDA-approved TKIs [30]. cMET is a protooncogene that encodes a protein known as hepatocyte growth factor receptor, whose abnormal activation in cancer correlates with poor prognosis. A cMET imaging radioligand is also under development that may be used to potentially identify cMET positive tumours prior to administration of a cMET inhibitor [31].

### 3.5. Indication of Normal Tissue Toxicity

PET studies can also provide crucial information on the differential uptake of the NME and its radiolabelled metabolites in various tissue organs. Although the presence of increased drug concentrations in normal tissue do not necessarily signify specific tissue toxicities, increased uptake, and retention in tissues such as the cardiac muscle, renal cortex, or the presence of enterohepatic circulation of radioactivity may alert to the need of a vigorous monitoring and management of potential normal tissue toxicities during drug development. Microdosing clinical studies with [^11^C]DACA, a drug that did not progress beyond phase II studies, demonstrated drug accumulation in the heart suggestive of a potential cardiotoxicity risk, which in the later phase II studies was fortunately not observed [32–34].

### 3.6. Drug Scheduling

PET studies may potentially help in the optimisation of drug scheduling strategies that are intended to maximize drug levels/exposure to the target tissue, while limiting exposure to healthy organs. Such studies can be conducted either during early or late drug development stages. By applying mathematical techniques to PET studies with [^11^C]temozolomide, it was possible to predict brain tumour and normal tissue temozolomide concentration profiles for different temozolomide dosing regimens [13]. This study suggested that the predicted biodistribution of temozolomide in tumour was better for the 200 mg/m^2^/day for 5 days q 28 days regimen compared to 100 mg/m^2^/day for 5 days q 28 days and 75 mg/m^2^/day for 7 weeks regimens, a finding consistent with current clinical practice.

### 3.7. Utility of PET Biodistribution Studies in Later Phase Clinical Development

Although in this paper we primarily focused on the utility of PET imaging in FHD or early proof of concept studies, information provided from such studies can also be helpful in later phase trials, in order to interrogate drug combinations, novel-novel-drug testing, schedule modification, and repositioning of the compound. Some of these studies are currently under way (Table 1).

## 4. Implementation Challenges for PET Imaging Exemplified by the FHD Study with Antisense Oligonucleotide (ASO) (LY2181308) Targeting the Apoptosis Inhibitor Protein Survivin

Having discussed the potential advantages of performing PET studies with radiolabelled NMEs in FHD studies, we shall now provide an overview of one such study illustrating the challenges during early cancer drug development. This is one of the few examples of an imaging study with a radiolabelled NME which was prospectively integrated in a FHD study [16].

### 4.1. Study Genesis and Aims

The compound, LY2181308 (Eli Lilly and Company), was radiolabelled because there was uncertainty on whether such an ASO would reach the tumour tissue. For such a biodistribution study, there were several advantages for an ASO and in particular for LY2181308: (a) the pharmacokinetics of the agent allowed a nearly full extrapolation from animals to humans [17]; (b) the manufacturing was well controlled with few impurities. Importantly, at the time of conception, there was neither pharmacokinetic nor biodistribution study data for any ASO performed in cancer patients; consequently additional information on distribution of the ASO to the tumour was needed prior to moving the NME into the next phase of clinical development. Therefore, normal tissue and tumour pharmacokinetics of this NME was determined and its antitumour effect in cancer patients was assessed.

### 4.2. Operational Setup and Study Organisation

Broadly, the process consisted of 3 main tasks (a) development of radiolabelling conditions to non-Good Manufacturing Practice (non-GMP) standards and performance of preclinical radiation dosimetry, (b) transfer of non-GMP radiochemistry to GMP standard, tracer validation for clinical use, and submission of a dossier detailing radiochemical manufacturing and quality control processes to the UK Medicines and Healthcare Regulatory Agency (MHRA) for approval, and (c) submission of the clinical study documents to the independent Research Ethics Committee (REC) and the Administration of Radioactive Substances Committee (ARSAC) for approval to conduct the study in cancer patients. This required multidisciplinary and multi-institutional collaborations between the Early Oncology Clinical Investigation Division at Eli Lilly and Company, the sponsor and owner of LY2181308, and the University of Washington, St. Louis, MO, USA (UW) for development of non-GMP radiochemistry and the University of Manchester Wolfson Molecular Imaging Centre, Manchester, UK (WMIC), for implementation of GMP radiochemistry and conducting the clinical study.

The initial step to perform feasibility evaluation of radiosynthesis of [^11^C]LY2181308 and development of non-GMP production at UW took over 15 months. The time period for this step is variable and will depend on the ease of introduction of the PET isotope. The synthetic route to label the NME often differs from that of the corresponding unlabelled drug since the short half-life of the isotope requires the radioisotope to be introduced as part of the final synthetic step. This means both precursor for labelling and the radiotracer may require development of their respective preparation route. Dosimetry studies were then conducted in nonhuman primates to estimate the human radiation effective dose [35]. Following this, technology transfer of the methods to WMIC was undertaken for implementation of GMP radiochemistry. Implementation of GMP radiochemistry and performance of validation runs were done to confirm the manufacturing and quality control processes of the tracer in accordance with the approved EU regulations. Validation runs were performed to ensure that the impurities and additives in the end-product were within acceptable levels for human administration and confirmed the sterility of the formulated [^11^C]LY2181308 doses produced. Although the formulated [^11^C]LY2181308 was going to be given to patients participating in the concurrent FHD study, the radioactive form of [^11^C]LY2181308 required a separate and updated Investigational Medicinal Product Dossier (IMPD) for this substudy of the FHD. This requirement was based on the fact that [^11^C]LY2181308 was prepared and formulated in a different way than the authorised drug product for the concurrent FHD study. The IMPD therefore included updates to the Chemistry, Manufacturing and Control (CMC) section. Medical sections had to also be updated in the IMPD for the FHD study to justify the PET biodistribution study.

### 4.3. Study Hurdles

**Figure 1:**
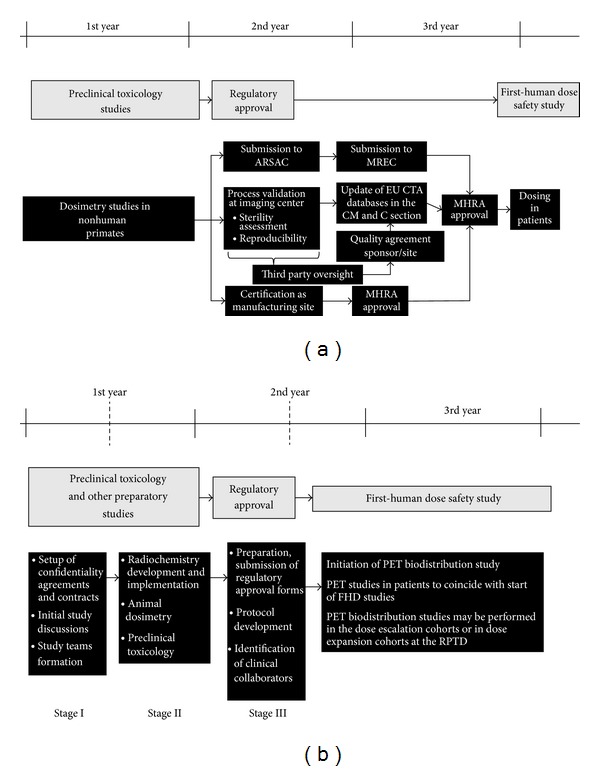
Summary of timelines and procedures performed in the PET biodistribution study of the ASO LY2181308 in relation to the FHD study (a). (b) Is proposed flowchart of future streamlined timelines of PET biodistribution studies in relation to FHD oncology studies with NMEs.

A number of considerable delays were encountered as this was the first of its kind study at that time (2005-2006) after introduction of the new regulatory processes emanating from the EU clinical trial directive. The major delay related to compiling adequate and sometimes novel documentation for regulatory review. In the light of new guidelines by the European Medicines Agency (EMEA) (EMEA 2006; [36]), a formal site inspection for assessing the manufacturing and production of the radiolabeled agent was performed by the UK Medicines and Healthcare products Regulatory Agency (MHRA) which added to the timelines. In parallel to the MHRA submissions other regulatory submissions to the Research Ethics Committee (REC) and the Administration of Radioactive Substances Advisory Committee (ARSAC) were made. Delays were also encountered with ethics approval as there were concerns about administration of radioactivity and the REC was unfamiliar with this type of study. Overall, the time from early radiochemistry work and dosimetry studies to the initiation of the clinical study took about 3 years (Figure 1).

### 4.4. Scientific Information Provided by the Study

Despite the long wait, this study provided valuable information, demonstrating rapid biodistribution of the radiopharmaceutical in tissues (including the tumour), an unanticipated but positive finding. In addition, greater than anticipated accumulation of [^11^C]LY2181308 was observed in the cortex of kidneys, a finding that differed from the preclinical data. Consequently, there was concern about ASO-related renal toxicity and this directed adequate monitoring strategies for phase II studies. Incidentally, the FHD study with LY2181308 [16, 17] included a patient in which the possible renal toxicity was of concern [37]; fortunately, this grade 2 toxicity was not dose-limiting and reversible. This unexpected finding during the biodistribution study shows another advantage of conducting such biodistribution studies early in drug development, because it detected an otherwise unappreciated toxicity risk. This study also demonstrated a decrease in tumour glucose metabolism as observed by a decrease in FDG-PET uptake, while simultaneously the target engagement was evaluated. Complementing the otherwise favourable safety profile, this concomitant tumour response and target engagement justified the advancement of LY2181308 into phase II development [38–40].

## 5. Current Outlook and Future Considerations

Early drug development is a dynamic process with a focus on timelines to ensure the delivery of NMEs to the next development phase. Radiolabelling of an NME is often commissioned late within a drug lifecycle and the impact of such data on key decisions can easily be lost. Delivery of a radiolabelled NME study needs to focus on providing the right data at the right time for the programme—and we argue that its greatest use is during early drug development.

Since the completion of the study with LY2181308, some of the processes have been positively streamlined in the UK. These include the implementation of the integrated research application system (IRAS) that allows for parallel submission to the various regulatory bodies including the Research Ethics Committee (REC), ARSAC, MHRA, and the hospital Research and Development (R&D) committees. Despite this, we feel that regulatory processes can be further streamlined in order to reduce timelines without sacrificing quality and patient's safety. For instance, although decisions from regulatory bodies such as REC, MHRA, and ARSAC are dictated by strict timelines, addressing multiple queries from RECs that either are unaware of or have not dealt with such biodistribution studies with radiolabelled NMEs may result in significant delays. Therefore, assignment of specialized RECs to review such PET studies would likely minimise delays.

In addition to the interaction with regulatory bodies, recruitment of patients to PET studies is dependent on the hospital R&D approval process, which is not time-bound. Discussions with the hospital R&D departments can lead to an impasse about study costs, extent of contractual obligations, intellectual property (IP) rights, and other legal interpretations of contracts. An early engagement with hospital R&D departments and the clinical investigators and/or mandating time-bound approval process may help reduce delays. The European Commission has also recognised that since the introduction of the EU Clinical Trials directive 2001/2/EC the number of clinical trial applications in the EU between 2007 and 2011 has fallen by 25%, whilst the costs for bureaucracy and resource requirements to handle paperwork have doubled, and delays have increased by 90%. The European Commission is therefore putting together new proposals that will reduce bureaucracy and include procedures to simplify and streamline the trial authorisation procedures. We hope that these proposals likely to come into effect in 2016 will improve the regulatory approval processes.

Currently PET clinical studies with radiolabelled NMEs are considered a clinical trial by the MHRA, requiring submission of an IMPD in the UK. As the MHRA now regularly audit imaging centres for PET studies, it is no longer a requirement that sites are reaudited before the initiation of every such biodistribution study. Guidelines for animal toxicity testing and for quality of all chemicals entering the manufacturing process and of the radiopharmaceutical (European commission GMP guidelines Annex 3, 2008 [41]) provide useful recommendations for the GMP implementation of radiopharmaceuticals for early phase clinical trials [42]. However, time and resources to conduct additional animal toxicology evaluation to enable PET imaging studies are minimal and consideration is given to include such studies in the standard animal toxicology evaluation if PET studies are planned in order not to add further time to the existing timelines.

Since animal dosimetry studies overestimate the radiation dose in humans and the radiation exposure for carbon-11 radiotracers is quite low (on average equivalent to about one year of background radiation exposure in the UK), we believe that dosimetry studies are not required, especially for carbon-11 labelled NMEs [43]. Even with Fluorine-18 radiolabelled radiotracers, the radiation exposure to the subject is on average similar to that obtained with a diagnostic FDG PET scan (or equivalent to about 3-year background radiation exposure) [44]; a case can be made for not performing animal dosimetry studies for radiolabelled NMEs, especially in a group of patients with limited life span and where the tracer is unlikely to be used as a diagnostic agent in the future.

Other processes may also lead to potential delays, including those related to transference of non-GMP to GMP methods. Radiolabelling feasibility of the NME and production of the radiopharmaceutical with good reproducibility may be highly variable. Hence, the feasibility evaluation process must consider the chemical structure and the ease of synthesis of the NME. The performance of the labelling feasibility work should be done on a similar automated platform as the one(s) to be used for the GMP implementation. This requires an appropriate PET radiochemistry infrastructure ideally with duplication of equipment, as R&D grade materials should not be used on equipment for GMP synthesis in accordance to EU guidelines. Involvement of the quality assurance department early in the development process will also make for an easy translation to the GMP settings. Finally, sourcing of a GMP-grade precursor, failure of validation runs, and delays in sterility analysis during validation may need to be addressed to avoid potential delays.

Therefore, good project management including a GMP implementation and risk management plan and regular interaction between the drug developers, radiochemists, and medicinal chemists during the development process will help focus on the timelines and address unforeseen issues. Project allocation to a team with expertise in R&D radiolabelling feasibility and translation to GMP will especially help avoid potential delays. Skilled leadership, effective communication and decision-making, formation of study teams consisting of members with the requisite skills, and identification of favoured suppliers to source precursors are likely to help prevent delays.

As the ultimate success of such studies is crucially dependent on patient recruitment, protocols should be patient-friendly. It is equally important that the small number of imaging centres work closely with the FHD study sites and the clinical investigators. Therefore, a well-established nexus of the imaging centre, phase I units, and clinical investigators that can deliver such studies will be attractive to the pharmaceutical industry.

On the basis of our experience, a period of up to 6 months is expected from initiation of a study proposal to confirmation of radiochemistry production feasibility. An additional 9 months is then required for the implementation of radiolabelling methods in accordance with the principles of GMP, regulatory submission, and approval until the initiation of the PET biodistribution study (Table 2). This period of 15 months for initiation of the PET study from study conception would fit well with the similar time required for standard initiation of a FHD clinical study from compound selection. We think that these streamlined timelines would allow for a greater integration of PET biodistribution studies during FHD clinical studies.

Despite the potential utility, anticipated improvements of organisational and regulatory interactions, and increased interest in radiolabelling NMEs [45], there is a missing collection of examples supporting the value for early clinical biodistribution studies. This lack of supporting information, perceived high costs, timelines, and overall complexity of the technology [46], in a current climate of tightening budgets, prevents us from fully assessing the impact of biodistribution studies on clinical development and hampers uptake by scientists and clinicians intimately involved in the drug development process. However, it is heartening to note that there are a few clinical PET biodistribution studies currently underway worldwide, as reflected in the NIH trial database (Table 1). While from this list it appears that NMEs are being evaluated in imaging studies, their value in the overall drug development is not clear. Nevertheless, we anticipate that results from such and other well-designed studies integrated into the drug development process will likely catalyse a wider use of this technology and an even greater focus on efficiency and timelines.

We would like to propose the following action points that would lead to greater implementation of such studies. Firstly, the key stakeholders from academia, industry, PET community, and imaging bodies should be brought together to act as a forum for discussion of proposals submitted by members who are new to this area. We envisage that this group would provide advice and guide in the setup and implementation of studies and also interact with the regulatory bodies. Because such specialized clinical imaging centres are limited, we would propose creating a network of accredited centres working closely with clinical investigators and clinical units that are able to implement all aspects of such studies. At the current stage, we do not expect more than 5 such studies to occur every year worldwide; however, this would likely increase if such studies prove their value in drug development.

## Figures and Tables

**Table 1:** Examples of some the clinical PET biodistribution studies performed with radiolabelled anticancer agents.

Drug	References
5-Fluorouracil	[10, 11]
Temozolomide	[12, 13]
N-[(2′-dimethylamino)ethyl]acridine-4-carboxamide (XR5000)	[14, 15]
Antisense oligonucleotide to Survivin (LY2181308)	[16, 17]
Docetaxel	[18]
Paclitaxel	[19]
Lapatinib	[20]
Erlotinib	[21–23]
Anti-CD44v6 chimeric monoclonal antibody, U36	[24]
Trastuzumab	[25, 26]
Some radiolabelled molecular entities under evaluation in ongoing imaging studies (NIH trial database)	
[^ 89^Zr]-Bevacizumab, [^89^Zr]-RO5323441 (placental growth factor antibody), [^18^F]-SKI-249380 (dasatinib derivative), [^124^I]-PUH71, MMOT0530A (a monoclonal antibody), [^18^F]-Paclitaxel, [^11^C]-Erlotinib, and [^89^Zr]-Cetuximab	

**Table 2:** Sequential stages of activities, their timelines, potential causes for delays, and recommendations to reduce delays in PET biodistribution studies.

Activities	Approx. time (months)	Potential causes for delays	Comments and recommendations for improvement in timelines
Stage I (all activities can be done simultaneously): time 1-2 months			

Initial study discussion formulation of an outline research plan	2 months	Prevarication, and absence of key decision makers	Clear communication and clear goal setting by the respective team leaders

Confidentiality agreements completion	1-2 months	Legal issues	Preexistence of a master services agreements

Formation of study teams with clear definition of roles and responsibilities	1 month	Organisational issues Availability of experienced staff and agreement	Ensure inclusion of key staff, chemists, regulatory, clinical, biologists

Contract agreements	1-2 months	Disagreements on pricing and intellectual property rights	Early finance and legal involvement. Work with contract-based imaging services providers

Stage II (all activities can be done simultaneously): time 4–7 months			

Radiochemistry feasibility and production of radiolabelled NME (non-GMP or GMP) Implementation of GMP radiochemistry (RC) and its validation Preclinical dosimetry study Animal toxicology studies	4–7 months	Radiochemical method development issues Issues with procurement of precursor (GMP or non-GMP) Problems transferring from non-GMP to GMP settings	Selection of PET centre with good track record of developing and implementing radiopharmaceuticals for clinical use and management of drug maker expectations Preferred approved suppliers Duplication of equipment for straightforward transfer of methods, development of cassette based chemistry, good communication, and regular interactions Agreement of clear milestones and associated timelines for “Go-No Go” decisions

Stage III (all activities can be done simultaneously): time 3–6 months			

Preparation of IMPD dossier for submission to MHRA	1 month	Subject to delays in GMP implementation	Experienced personnel to complete CMC section

Identification of clinical collaborators Initiation of contracts with hospital	3 months	Identification and selection of appropriate clinicians Realistic assessment of recruiting potential	Initiate early in Stage III as can cause significant delays Preferred clinicians, imaging centre-linked clinical units

Development of research study protocol	2 months	Internal hierarchical and regulatory approval	

Preparation and submission of regulatory submission to the REC, MHRA, and ARSAC and to hospital R and D	2 months	CMC section completion due to RC implementation delays Delays in getting a date for an REC meeting	Done via the common IRAS However, MHRA and ARSAC require additional information to that provided by IRAS

Obtaining regulatory approvals and study initiation	2-3 months	Delays if there are queries or need for modifications Variable calibre of ethics committee to review such studies	Hospital R and D approval not time-bound Submission to or designation of ethics committees to review such studies
